# Missouri Valley Veterinary Medical Association

**Published:** 1895-12

**Authors:** S. L. Hunter

**Affiliations:** Secretary; Fort Leavenworth, Kan.


Secretary.

### MISSOURI VALLEY VETERINARY MEDICAL ASSOCIATION.

THE sixth regular meeting was held in Kansas City, October 2, 1895. It was called to order at 7.30 P. M. by President S. Stewart, after making an appropriate address. Roll-call was answered by the following members: Drs. Ernst, Sihler, Wattles, Forbes, Mayo, Biart, Thompson, Cock, Hill, King, Topping, C. O. Netherton, J. B. Black, and S. L. Hunter. Others present were Drs. Rowell, Liberty, Mo.; Verschelden, St. Mary's, Kan. McClellan, Lawrence, Kan.; Eisenhower, Abilene, Kan.; J. Wilson, St. Joseph, Mo.; Albert J. Payne, Kansas City, Mo.; Mr. Hadley, a prominent stockman of Lawrence, Kan., and students of the Kansas City Veterinary College, and others.

The papers presented were all well prepared, and the discussion excellent.

Dr. Forbes, of St. Joseph, Mo., read a valuable paper entitled "Observations in Tuberculosis;" Dr. Biart, of Lansing, Kan., a paper on "That Glanders is more Contagious than Infectious;" Dr. B. F. Kaupp on "Gastro-entèritis, a Sequel of Flatulent Colic produced by Acute Indigestion." A number of interesting cases were described, and the discussions were spirited and enjoyed by all.

The following applicants were elected to membership: Drs. Payne, Kansas City, Mo.; McClellan, Lawrence, Kan.; Verschelden, St. Mary's, Kansas.

Adjourned to meet in Kansas City, Mo., in December.

S. L. HUNTER, V.S.,

Secretary.

FORT LEAVENWORTH, KAN., Nov. 1, 1895.

